# Estradiol Modulates Membrane-Linked ATPases, Antioxidant Enzymes, Membrane Fluidity, Lipid Peroxidation, and Lipofuscin in Aged Rat Liver

**DOI:** 10.4061/2011/580245

**Published:** 2011-10-09

**Authors:** Pardeep Kumar, R. K. Kale, Najma Zaheer Baquer

**Affiliations:** School of Life Sciences, Jawaharlal Nehru University, 110067 New Delhi, India

## Abstract

Free radical production and oxidative stress are known to increase in liver during aging, and may contribute to the oxidative damage. These changes increase during menopausal condition in females when the level of estradiol is decreased. The objective of this study was to observe the changes in activities of membrane linked ATPases (Na^+^K^+^ ATPase, Ca^2+^ ATPase), antioxidant enzymes (superoxide dismutase, glutathione-S-transferase), lipid peroxidation levels, lipofuscin content and membrane fluidity occurring in livers of female rats of 3, 12 and 24 months age groups, and to see whether these changes are restored to 3 months control levels rats after exogenous administration of 17-**β**-estradiol (E2). The aged rats (12 and 24 months) were given subcutaneous injection of E2 (0.1 **μ**g/g body weight) daily for one month. The results obtained in the present work revealed that normal aging was associated with significant decrease in the activities of membrane linked ATPases, antioxidant enzymes, membrane fluidity and an increase in lipid peroxidation and lipofuscin content in livers of aging female rats. The present study showed that E2 treatment reversed the changes to normal levels. E2 treatment may be beneficial in preventing some of the age related changes in the liver by increasing antioxidant defenses.

## 1. Introduction

Free radical production and oxidative stress are known to increase in liver during aging and may contribute to the oxidative damage, which plays an important role in the aging process [1]. Oxidative stress has been implicated in the pathogenesis of several alterations due to menopause and can arise through the increased production of lipid peroxides and/or a deficiency of antioxidant defense [2, 3]. 

Recently, there has been a growing interest in the actions and functions of the estrogen replacement therapy and steroid hormone estrogens (17-*β*-estradiol, E2) important role in the progression of chronic hepatic diseases and synaptic plasticity [4, 5]. Moorthy et al. [6] reported that older women might also benefit from the protective effects of hormone replacement therapy (HRT) that uses a relatively lower concentration of hormones. E2 could enhance antioxidant and antiapoptotic activity in hepatic fibrosis in rats [7, 8]. 

Morphofunctional studies suggest that the liver, compared with other organs, ages fairly well [9]. Superoxide dismutase (SOD), which decreased in rat liver, brain, heart, kidney, and uterus [10, 11] during aging, constitutes an important defense system to clear up the detrimental reactive oxygen species (ROS) *in vivo* [2]. Glutathione S-transferases (GST) belong to a group of multigene and multifunctional detoxification enzymes, which defend cells against a wide variety of toxic insults from chemicals, metabolites, and oxidative stress [12]. GST decreased with aging in liver [13], brain [14], and heart [11]. 

Oxidative stress is postulated to be one of the most important mechanisms behind age-related decrease in SOD and GST activities in aging rats [15, 16]. Accordingly, many studies in the past attempted to clarify how these enzyme activities are altered during aging (for review, see [2, 17]).

Na^+^K^+^ ATPase and Ca^2+^ ATPase are transmembranous enzymes that play an important role in maintaining ionic homeostasis and physiologic function of the liver [18, 19]. Decreased Na^+^K^+^ ATPase and Ca^2+^ ATPase activities in liver of aging animals affect the signal transduction pathway and cellular functions, suggested as a contributing factor in the development of age-related disorders with increase in the content of lipid peroxidation [20, 21]. 

Free radicals oxidize polyunsaturated fatty acid leading to the fact that membrane fluidity decreases with aging, impairing the crucial membrane functions of transport and permeability [22, 23]. Malondialdehyde (MDA) is one of the end products in the lipid peroxidation process which leads to increase in phospholipids rigidity [24]. Lipofuscin is an autofluorescent pigment that accumulates inside aging tissue due to increased oxidative stress which represents an end product of oxidative degradation of lipids [25, 26]. Kumar et al. [27, 28] had shown that lipofuscin is a peroxidation product, and its formation appears to be proportional to the occurrence of lipid peroxidation.

The aim of the present study was to investigate the antiaging and protective potential of lower doses of E2 than HRT levels treatment on activities of membrane-linked ATPases (Na^+^K^+^ ATPase, Ca^2+^ ATPase), antioxidant enzymes (SOD, GST), lipid peroxidation levels, lipofuscin content, and membrane fluidity in the liver of aging female rats.

## 2. Materials and Methods

### 2.1. Animals

Female albino rats of the Wistar strain of different ages namely 3, 12, and 24 months (*n* = 8 for each group) were used for all the experiments. Animals were maintained in the animal house facility of Jawaharlal Nehru University (JNU), New Delhi, India, at a constant temperature of 25°C, humidity of 55% at 8:00 to 20:00 h light, and 20:00 to 8:00 h dark cycle. The animals were fed standard chow (Hindustan Lever Ltd., India) and given tap water ad libitum until treatment or time of sacrifice. All the animal procedures were approved by the Institutional Animal Ethical Committee (IAEC) of Jawaharlal Nehru University, New Delhi, India

### 2.2. Hormone Administration

The aged rats (12 and 24 months old) (*n* = 8 for each group) were given subcutaneous injection of 17-*β*-estradiol (0.1 *μ*g/g body weight) daily for one month. E2 was dissolved in propylene glycol in appropriate concentrations [6, 29]. Control animals received an equal volume of vehicle. There was no treatment on the day of the sacrifice. After 30 days of hormone treatment, experimental animals of all the groups were sacrificed and livers were isolated for further study.

### 2.3. Preparation of Homogenates and Subcellular Fractions

Animals were sacrificed by cervical dislocation. Livers were rapidly excised and washed with chilled normal saline. The homogenates and subcellular fractions were prepared as described earlier; the pellet obtained at 12,000 g containing mitochondria and the supernatant fractions were used for enzyme assays [6, 30]. The pellet was used for determining Na^+^K^+^ ATPase and Ca^2+^ ATPase activities and membrane fluidity. Whole homogenates were used for measurement of lipofuscin and lipid peroxidation. Supernatant fraction was used for estimation of GST and SOD activities.

### 2.4. Measurements of Enzymes

#### 2.4.1. Assay of SOD (EC: 1.15.1.1)

The activity of SOD was determined by the method of S. Marklund and G. Marklund [31], using inhibition of pyrogallol autooxidation at Ph 8.0. The specific activity of SOD is expressed as units per mg protein per minute. 

#### 2.4.2. Assay of GST (EC: 2.5.1.1.8)

The specific activity of cytosolic GST was determined spectrophotometrically at 37°C as described by Gupta et al. [32]. The reaction mixture (1 mL) contained final concentration of 0.1 M phosphate buffer (pH 6.5), 1 mM CDNB in 95% ethanol, and 1 mM GSH and was incubated at 37°C for 5 min. The specific activity of GST is expressed in terms of micromoles of GSH-CDNB conjugate formed/min/mg protein.

#### 2.4.3. Assay of Na^+^K^+^ ATPase (E.C: 3.6.1.37)

Na^+^K^+^ ATPase activity was measured in the pellet according to the method of Mayanil et al. [33]. The enzyme activity was calculated as the difference of the activity between total ATPase and Mg^2+^ ATPase. The specific activity of the enzyme is expressed as *μ*mol Pi released/mg protein/min. 

#### 2.4.4. Assay of Ca^2+^ ATPase (EC: 3.6.3.8)

Ca^2+^ ATPase activity was measured in the pellet, using a colorimetric method described by Desaiah et al. [34]. The pellet (~50 *μ*g mitochondrial protein) was incubated in the reaction medium of final volume 1 mL. The specific activity of the enzyme is expressed as *μ*mole Pi released/mg protein/min.

### 2.5. Measurement of Lipid Peroxidation

The formation of lipid peroxides was measured in the homogenates of the whole liver. The formation of MDA, an end product of fatty acid peroxidation was measured spectrophotometrically at 532 nm by using a thiobarbituric acid reactive substance (TBARS) essentially by the method of Genet et al. [35]. Results are expressed as nmole of MDA formed/mg protein. 

### 2.6. Lipofuscin Content

The extraction of lipofuscin and measurement of its fluorescence were essentially as described by Tappel et al. [36]. Using a primary filter (366 nm) for excitation and a secondary filter for emission (485 nm) fluorescence measurements were carried out using a SLM 4800 model spectroflourometer. Quinine sulphate (0.1 *μ*g/mL) in 0.05 M H_2_SO_4_ was used as a standard. The results were expressed as percentage fluorescence of control from 1 mL of homogenate (w/v). 

### 2.7. Membrane Fluidity

The pellets were labelled with 1,6-diphenyl-1, 3, 5-hexatriene (DPH), a fluorescent probe by incubating equal volume of the suspension containing 100 *μ*g·mL^−1^ of protein in phosphate buffer and 2 *μ*M DPH suspension in the same buffer. Excitation and emission wavelengths were, respectively, 365 and 428 nm. Polarization (*P*) and anisotropy (*r*) measurements were carried out on a model SLM 4800 polarization spectroflourometer as described by Kumar et al. [29].

### 2.8. Protein Estimation

Protein was estimated in subcellular fractions by the method of Bradford [37] using bovine serum albumin (BSA) as standard.

### 2.9. Statistical Analysis

Results were analyzed using Prism 5.0 (GraphPad, San Diego, Calif, USA). The values are expressed as mean ± SEM of 4–6 separate values. The data were analyzed using one-way ANOVA followed by Turkey-Kramer multiple comparison test to determine the statistical significance. The significance was calculated by comparing the age-matched controls with experimental groups.

### 2.10. Chemicals

All purified enzymes, coenzymes, substrates, standards, and buffers were from Sigma Chemicals Company, USA. All other chemicals were of analytical grade and purchased from SRL and Qualigens, India. 

## 3. Results

### 3.1. Effect of E2 on General Physiological Parameters in Aging

An increase in body weight throughout the study was observed with aging in all the groups. The body weights of the aging animals increased significantly (*P* < 0.05) at 12 and (*P* < 0.001) in 24 months when compared with 3-month control rats. There was a significant decrease (*P* < 0.05) with E2 treatment in 24-month age groups when compared with respective controls. There were no significant changes in body weight at 12- and 24-month E2 treatment groups when compared with 3-month control rats. The protein content in the whole homogenates, supernatant, and pellet fractions did not show significant changes with aging and E2 treatment. Results are shown in the Table 1.

### 3.2. Effect of E2 on Antioxidant Status in Aging

**Figure 1:**
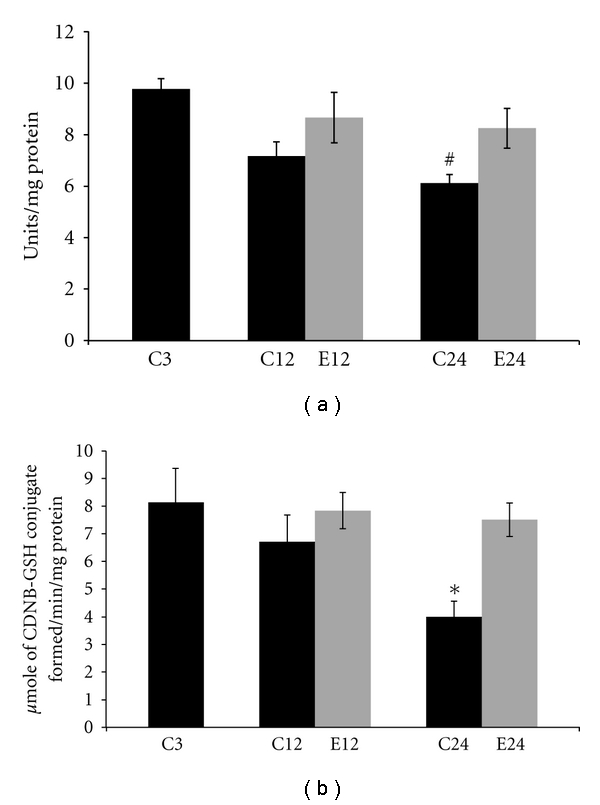
Changes in the activities of antioxidant enzymes (a) superoxide dismutase (SOD) and (b) glutathione S-transferase (GST) in liver supernatant fraction of 3, 12, and 24 months of control (C) and estradiol- (E2-) treated aging female rats. Each value is a mean ± SEM of five or more separate values from two to three experiments. The comparisons of experimental values are with the control values. Statistical significance: ^#^ *P* < 0.01, *P < 0.05 versus 3 months.

Changes in the activities of SOD and GST in cytosolic fractions in liver of 3, 12, and 24-month control and E2-treated aging female rats are summarized in Figure 1. With aging there was a decrease in SOD activity compared to 3-month control animals. In 24-month control group a significant (*P* < 0.01) decrease was seen in SOD activity when compared with 3-month controls. Treatment of E2 to aging animals increased SOD activity in 12 and 24 months, when compared with respective age-matched controls. There was no significant change in SOD activity at 12- and 24-month E2 treatment groups when compared with 3-month controls. 

With aging there was a decrease in GST activity as compared to 3-month control animals. In 24 months there was a significant (*P* < 0.05) decrease in GST activity when compared with 3-month controls. When compared with respective age control group, an increased GST activity at 12- and 24-month E2-treated animals was seen. Treatment with E2 to aging rats restored the antioxidant enzyme activity nearly to 3-month controls. There was no significant change in GST activity at 12- and 24-month E2 treatment groups when compared with 3-month control rats.

### 3.3. Effect of E2 on Membrane-Linked ATPases in Aging

**Figure 2:**
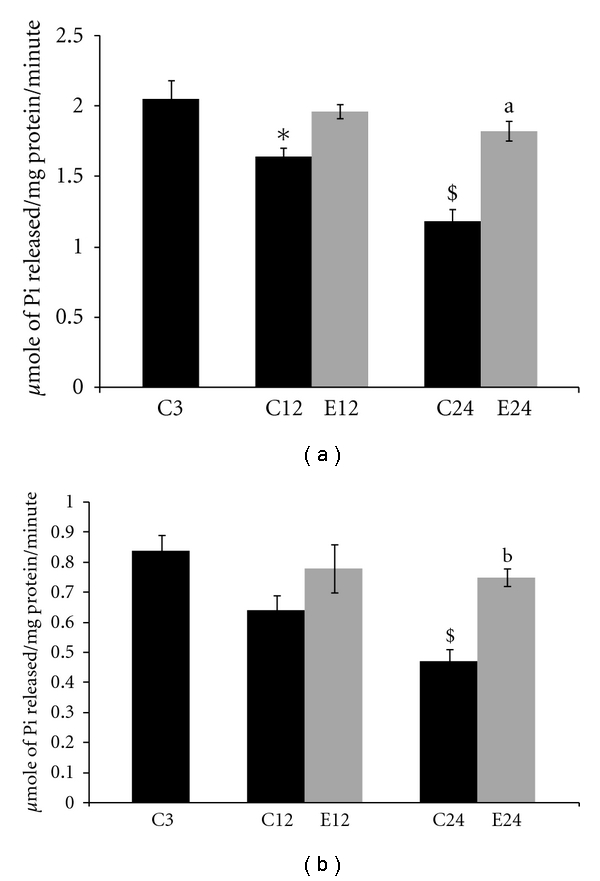
Changes in the activities of membrane-linked ATPase (a) Na^+^K^+^ ATPase and (b) Ca^2+^ ATPase in liver mitochondria of 3, 12, and 24 months of control (C) and estradiol- (E2-) treated aging female rats. Each value is a mean ± SEM of five or more separate values from two to three experiments. The comparisons of experimental values are with the control values. Statistical significance: ^a^ *P* < 0.001, ^b^ *P* < 0.01 comparing age-matched controls versus E2 treatment and ^$^ *P* < 0.001,*P < 0.05 versus 3 months.

Changes in the activities of Na^+^K^+^ ATPase and Ca^2+^ ATPase in pellet fractions of 3, 12, and 24-month control and E2-treated aging female rats are summarized in Figure 2. With aging there was a decrease in Na^+^K^+^ ATPase activity as compared to 3-month controls. In 12- and 24-month control group, there was a significant (*P* < 0.05) and (*P* < 0.001) decrease in Na^+^K^+^ ATPase activity when compared with 3-month controls, respectively. When compared with respective age controls group, there was no significant changes in 12-month E2-treated animals. In 24-month aged rats Na^+^K^+^ ATPase activity increased significant after the E2 treatments (*P* < 0.001) when compared with respective age-matched controls. There was no significant change in Na^+^K^+^ ATPase activity at 12- and 24-month E2 treatment groups when compared with 3-month control rats.

With aging there was a decrease in Ca^2+^ ATPase activity as compared to 3-month control animals. In 24 months there was a significant (*P* < 0.001) decrease in Ca^2+^ ATPase activity when compared with 3-month controls. When compared with respective age controls, there was no significant change in 12-month E2-treated animals. In 24-month aged rats Ca^2+^ ATPase activity increased after the E2 treatments (*P* < 0.01) as compared to age-matched controls. There was no significant change in Ca^2+^ ATPase activity at 12- and 24-month E2 treatment groups when compared with 3-month rats. Treatment with E2 in aging rats restored the membrane-linked ATPases activities nearly to 3-month controls.

### 3.4. Effect of E2 on Membrane Lipid Peroxidation in Aging

**Figure 3:**
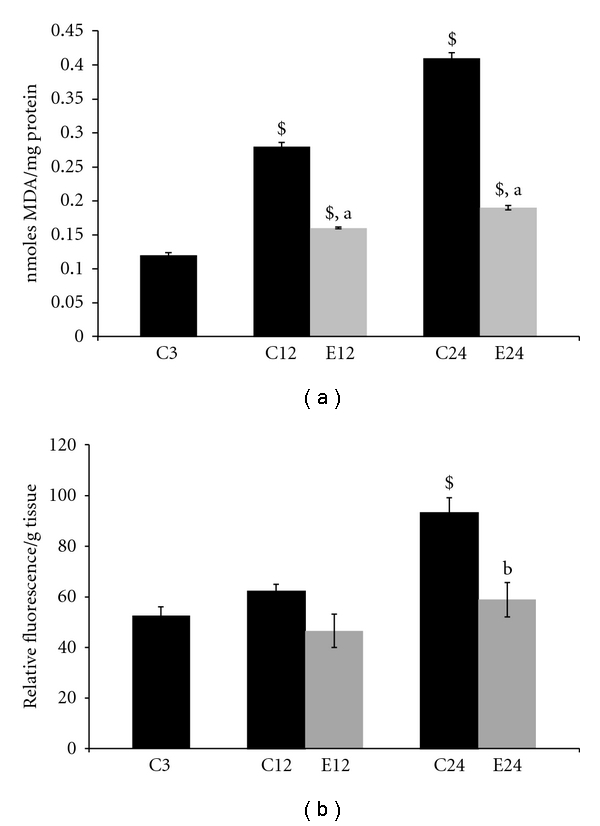
Changes in lipid peroxidation (a) and lipofuscin content (b) in liver of 3, 12, and 24 months of control (C) and estradiol- (E2-) treated aging female rats. Each value is a mean ± SEM of five or more separate values from two to three experiments. The comparisons of experimental values are with the control values. Statistical significance: ^a^ *P* < 0.001, ^b^ *P* < 0.01 comparing age-matched controls versus E2 treatment and ^$^ *P* < 0.001 versus 3 months.

Lipid peroxidation was measured as the formation of MDA in whole homogenates of aging rat liver from control and E2-treated aging animals. With aging there was significant increase (*P* < 0.001) in MDA levels at 12- and 24-month age groups as compared to the 3-month control animals. For E2 treatment, to 12 and 24 months old, the MDA levels decreased significantly (*P* < 0.001) at 12 months and (*P* < 0.001) in 24-month rats, respectively, when compared with age-matched controls. There was a significant (*P* < 0.001) change in MDA levels at 12- and 24-month E2 treatment groups when compared with 3 months. Administration of E2 restored the MDA levels nearly to 3-month controls. Results are shown in Figure 3(a).

### 3.5. Effect of E2 on Lipofuscin Content in Aging

Lipofuscin content was measured in whole homogenates of aging rat liver of control and E2-treated aging animals. With age there was significant increase (*P* < 0.001) in lipofuscin content at 24 months as compared to the 3-month control animals. Administration of E2 treatment decreased lipofuscin content in 24 months, respectively, as compared to the age-matched controls (*P* < 0.01). There was no significant change in lipofuscin content at 12- and 24-month E2 treatment groups when compared with 3-month control rats. Results are shown in Figure 3(b).

### 3.6. Effect of E2 on Membrane Fluidity in Aging

The DPH fluorescence polarization (*P*) and anisotropy (*r*) values were found to increase (*P* < 0.001) in the liver pellet with aging, reflecting a decrease in the probe mobility and an increase in membrane structural order or a decrease in membrane fluidity. The membrane fluidity is inversely related to the anisotropy and polarization parameters. The changes in fluidity measured in control and E2-treated animals of different age groups showed significant decrease in fluidity with increasing age from 3 months to 24 months. Twelve and 24-month E2-treated groups elicited a significant (*P* < 0.01) and (*P* < 0.001) decrease in anisotropy as compared to the respective controls. There was a significant (*P* < 0.01) increase in polarization values at 12-month E2 treatment groups when compared with 3-month control rats. Results are presented in Table 2. 

## 4. Discussion

Free radical production and oxidative stress are known to increase during aging and may contribute to the oxidative damage, which plays an important role in the aging process [1]. The impairment of liver metabolism and its gross architecture, during aging, finally leads to liver cell death [9]. Age-related changes in liver may have important implications for systemic immune responses and hepatic function [38]. Liver tissue has the highest population of macrophages in the body and therefore has a great potential for producing local and/or systemic inflammatory responses [39]. Recently Kireev et al. [40] had demonstrated that aging produced significantly increased levels of the proinflammatory cytokines TNF-a, IL-1b, and IL-6, whereas the anti-inflammatory IL-10 showed a decrease in liver homogenates of old female rats. Administration of the E2 treatment inhibits the induction of proinflammatory cytokines and iNOS, decreasing the levels of oxidative stress markers [41].

The present study elucidated the antiaging and protective potential of E2 treatment on activities of membrane-linked ATPases (Na^+^K^+^ ATPase, Ca^2+^ATPase), antioxidant enzymes (SOD, GST), lipid peroxidation levels, lipofuscin content, and membrane fluidity in the livers of aging female rats. Earlier data from our laboratory showed decreased levels of E2 at 12 and 24 months as compared to the 3-month cyclic rats [10]. The body weight of the female rats increased with age, primarily due to the increased amount of adipose tissue in the aged rats. A significant decrease in body weight was found with E2 treatment, which could be due to the antiobesity action of E2 [6]. The liver weights were also increased with age when compared to control (3 months old) animals. After the E2 treatment the liver weight was increased. This increase may be due to increased level of protein content in the liver (data not shown). Protein content of the hormone-treated rats which increased significantly in liver may be due to decreased protein catabolism and increased protein synthesis and also due to decreased gluconeogenesis [6, 25].

The present study reiterates the age-related decline in activities of antioxidant enzymes in aging liver. Present data showed that SOD activity decreased significantly in liver of aged animals, agreeing with earlier reports [10, 16, 42]; results also show a parallel decline in GST activity with aging [20, 43]. E2 treatment to aging rats, which restored SOD and GST activities, may be due to a decrease in free radicals generation by E2 and increased in antioxidant defenses [5, 44]. Kireev et al. [45] showed that ovariectomy decreased GST activity, increased mitochondrial damage in liver, and these changes were improved by E2 administration. Several studies by the group of Viña et al. [46, 47] showed the role of E2 in different organs, including liver. Estrogen administration regulates the expression of antioxidant, longevity-related genes, and consequently oxidant levels in aging animals. Estrogens modulate the antioxidant gene expression via the transcription factor as NF-kB. Estrogens upregulate mitochondrial antioxidant genes in females, leading to a lower mitochondrial oxygen radical generation, and help in longer longevity of females versus males in Wistar rats [48]. 

Na^+^K^+^ ATPase activity decreased significantly in liver mitochondria of aged animals when compared to 3-month control animals. This is in agreement with earlier studies [20, 42]. The decrease in Na^+^K^+^ ATPase could be due to increased oxidative stress and ROS formation with aging leading to increased lipid peroxidation and further damage to membrane structure. E2 treatment to aging animals restored the Na^+^K^+^ ATPase activity to almost control levels. E2 may be acting as a radical scavengers by decreasing ROS in aged rats and maintain the fluidity [4, 49]. In the present study, Ca^2+^ ATPase activity decreased in liver mitochondria of aging rats when compared with 3-month controls. The decreased Ca^2+^ ATPase activity may lead to increase in intracellular Ca^2+^ levels, alter the cellular fluidity, and eventually result in cell death [36]. E2 treatment to aging rats prevented the decreased Ca^2+^ ATPase activity, also decreased oxidative stress and ROS in aged rats, and restored the membrane fluidity. E2 increased membrane-linked ATPases; perhaps E2 acts as an endogenous modulator of ATPases and attenuates the impact of age-related Ca^2+^ dyshomeostasis [10, 49]. 

With aging there is significant increase in lipid peroxidation at 12- and 24-month old rats as compared to 3-month controls. Ample evidence showed that peroxidative damage to lipid and protein occurs with the aging process and that the products of these reactions accumulate with age [2, 27, 28, 50, 51]. Aging rats treated with E2 reversed the MDA levels to almost normal levels in the liver, suggesting its anti-lipidperoxidative abilities [8, 49]. Lipofuscin is a peroxidation product, and its formation appears to be integrative and proportional to the occurrence of lipid peroxidation. An age-related increase in lipid peroxidation has been shown to correlate with the gross level of lipofuscin [49]. E2 treatment to aging animals showed a decrease in lipofuscin content in liver [4, 29, 52]. 

Present results showed the age-dependent reduction of membrane fluidity [29, 53]. The treatment of aging animals with E2 reverted the membrane fluidity to normal level as compared to untreated age-matched controls. E2 treatment decreased free radicals formation and decreased lipid peroxidation levels, which further improves membrane fluidity [54]. Estrogen has been known to function as a radical scavenger inhibiting lipid peroxidation *in vivo* and *in vitro* [2, 10, 29]. 

E2's beneficial effects seemed to arise from its biochemical and metabolic effects as an antioxidant and protective role. It can therefore be concluded that age-related effects by estrogen hold great promise for improving the clinical management of selected age-related diseases and metabolic syndrome. 

## Figures and Tables

**Table 1:** Body weight and protein concentration of 3, 12, and 24 months of control (C) and estradiol- (E2-) treated aging female rats.

Age/treatment	Body wt. (g)	Protein content (mg/g)		
		Whole homogenate	Supernatant	Mitochondria
3 months				
C	218 ± 23	168 ± 17.3	129 ± 9.3	17.9 ± 1.6

12 months				
C	330 ± 18*	160 ± 18.5	110 ± 4.9	19.5 ± 2.1
E2	243 ± 27	164 ± 7.9	118 ± 8.6	20.8 ± 2.7

24 months				
C	425 ± 36^$^	156 ± 13.6	108 ± 7.4	17.2 ± 1.4
E2	292 ± 28^c^	162 ± 8.3	120 ± 4.5	18.6 ± 1.2

Each value is a mean ± SEM of five or more separate values from two to three experiments. The comparisons of experimental values are with the control values. Statistical significance: ^c^ *P* < 0.05 comparing age-matched controls versus E2 treatment and ^$^ *P* < 0.001, *P < 0.05 versus 3 months.

**Table 2:** Changes in polarization and anisotropy measurements in liver of 3, 12, and 24 months of control (C) and estradiol- (E2-) treated aging female rats.

Age/treatment	Membrane fluidity	
	Polarization (*P*)	Anisotropy (*r*)
3 months		
C	0.170 ± 0.002	0.157 ± 0.002

12 months		
C	0.208 ± 0.003^$^	0.195 ± 0.003^$^
E2	0.195 ± 0.002^#^	0.172 ± 0.004^b^

24 months		
C	0.224 ± 0.008^$^	0.203 ± 0.005^$^
E2	0.179 ± 0.002^a^	0.161 ± 0.008^a^

Each value is a mean ± SEM of five or more separate values from two to three experiments. The comparisons of experimental values are with the control values. Statistical significance: ^a^ *P* < 0.001,^b^ *P* < 0.01 comparing age matched controls versus E2 treatment and ^$^ *P* < 0.001, ^#^ *P* < 0.01 versus 3 months.

## Abbreviations

E2: 17-*β*-estradiol,
GST: glutathione S-transferases,
HRT: hormone replacement therapy,
MDA: Malondialdehyde,
ROS: reactive oxygen species,
SOD: superoxide dismutase.
